# Effects of Sphingomyelin on Skin Conditions in Healthy Adults: A Randomized, Double-Blind, Placebo-Controlled Trial

**DOI:** 10.7759/cureus.104321

**Published:** 2026-02-26

**Authors:** Shutaro Kubo, Hirotsugu Oda, Takashi Koikeda, Miyuki Tanaka

**Affiliations:** 1 Food Function Research Institute, Morinaga Milk Industry Co. Ltd., Zama, JPN; 2 Dermatology, Shiba Palace Clinic, Tokyo, JPN

**Keywords:** ceramide-phosphocholine, milk ceramide, skin barrier function, skin hydration, sphingomyelin

## Abstract

Background

Sphingomyelin (Sph) is a bioactive phospholipid, and its ingestion is suggested to improve skin conditions. Dairy products are accessible dietary sources of Sph. We investigated the effects of dairy-derived Sph on skin conditions.

Methods

A randomized, double-blind, placebo-controlled trial was conducted. The participants were healthy adult women aged 30-59 years recruited in Japan, who ingested a tablet containing dairy-derived Sph (23.6 mg/day) or a placebo tablet for 12 weeks. The primary endpoints were skin hydration and transepidermal water loss (TEWL). The secondary endpoint was the subjective perception of skin hydration.

Results

Overall, 94 participants were enrolled and randomly assigned to two groups (placebo, n = 48; Sph, n = 46). At the end of the intervention, 84 participants remained, and 83 (placebo, n = 44; Sph, n = 39) were analyzed. The increase in skin hydration levels on the upper arm in the Sph group was higher than that in the placebo group. TEWL and subjective perception of skin hydration showed no significant differences between the two groups. The incidence of adverse events was comparable between groups, and no adverse drug reactions were found.

Conclusion

Intake of dairy-derived Sph is safe and improves skin hydration. Sph-rich dairy products can be used to maintain skin health.

## Introduction

The skin is the largest organ in the body and plays a critical role in maintaining homeostasis. The outermost layer of the skin is the epidermis, which consists of four layers: stratum corneum (SC), stratum granulosum, stratum spinosum, and stratum basale, in order from the outside [1,2]. Among these, the SC is particularly important for maintaining skin barrier function, including moisture retention and protection against the external environment [3]. The SC originates from the stratum basale and undergoes progressive differentiation through the stratum spinosum and stratum granulosum [4]. The general turnover cycle is approximately six to eight weeks [5,6], though it is longer in areas where the epidermis is thicker [7]. The SC is mainly composed of corneocytes and intercellular lipids [8]. The major component of lipids is ceramide, which forms a lamellar structure in which ceramides line up regularly, holding water. Notably, this structure is a source of the skin barrier function [9]. Ceramides are synthesized in the stratum granulosum by ceramide synthase [10]. When structures are damaged or ceramides are deficient, skin barrier function declines, leading to increased water evaporation and dry skin, which causes skin problems [3,4,9]. Therefore, transepidermal water loss (TEWL) and skin hydration levels are generally used as indicators of skin barrier function and skin condition [11]. As causes of disruption of the barrier function, environmental stressors, such as low humidity and irradiation of ultraviolet (UV), are well known [12,13]. Recent studies have reported that mental stressors also disrupt the barrier function [14].

Sphingomyelin (Sph) is a bioactive phospholipid, and dairy products are major and accessible sources of dietary Sph. In cow’s milk, Sph is abundant in milk fat globule membranes (MFGM) [15]. Therefore, some unique compositions containing dairy-derived Sph have been applied as functional food ingredients [16]. Regarding the molecular structure of Sph, it consists of ceramides and choline phosphate. Ceramides themselves are composed of a sphingoid base and a fatty acid [17]. Due to its structural relationship to ceramides, Sph is sometimes referred to as ceramide-phosphocholine [18]. Accordingly, Sph-containing ingredients derived from cow’s milk are occasionally termed as “milk ceramides” [19,20]. Ingested Sph is digested and degraded into its components and is suggested to be absorbed in the intestine [21]. Importantly, low concentrations of sphingoid bases upregulate ceramide synthesis in cultured normal human epidermal keratinocytes [22]. Moreover, the oral administration of Sph derived from porcine or bovine to mice improves skin barrier function [20,22]. The intake of glucosylceramides derived from various plants containing sphingoid bases in the molecular structure improves skin conditions [23-25]. These data suggest that the intake of Sph improves skin barrier function in humans by upregulating ceramide synthesis in the epidermis. In particular, dairy-derived Sph has a history of dietary consumption with various food formats and is regarded as safe. However, the effects of common dairy-derived Sph on skin conditions have not been adequately investigated through rigorous study designs. In this study, we investigated the effects of oral intake of dairy-derived Sph on TEWL and skin hydration levels, along with subjective sensation of skin hydration in healthy adult women through a randomized, double-blind, placebo-controlled trial design.

## Materials and methods

Trial design and ethical approval

A randomized (1:1), double-blind, placebo-controlled, parallel-group comparative superiority trial was conducted in Japan by Shiba Palace Clinic (Tokyo, Japan) between December 2023 and March 2024. This trial was carried out in compliance with the latest version of the Declaration of Helsinki [26] and the ethical guidelines for medical and health research involving human participants [27]. The study protocol was approved by the Shiba Palace Clinic Ethics Review Committee on October 26, 2023 (approval no. 152240-35088) and registered with the UMIN Clinical Trials Registry on November 23, 2023 (registration no. UMIN000052879). The registration can be viewed at https://center6.umin.ac.jp/cgi-open-bin/ctr_e/ctr_view.cgi?recptno=R000060338.

Participants

Healthy adult women aged 30-59 who experienced dry skin and temporary work-related stress were recruited in Japan by SOUKEN Co., Ltd. Individuals meeting any of the following exclusion criteria were excluded from participation: (1) participants having a milk allergy; (2) participants whose lifestyle or living environment is possibly to change significantly during the study; (3) participants whose bedtime and/or waking time may change significantly during the study; (4) participants who are receiving invasive medical treatments or spa treatments impacting measured skin locations; (5) participants who have participated in other clinical trials within the past 3 months; (6) participants who are pregnant or under lactation, or who are expected to be pregnant during the study; (7) participants who have serious disease in the liver, kidney, heart, lung, gastrointestinal tract, blood, endocrine system, metabolic system, or have chronic disease etc. or those who have serious medical history of these; (8) participants who have skin diseases, mental illness, a sleep disorder, or a medical history; (9) participants who need to be on medication for allergic diseases such as pollinosis; (10) smokers; (11) extremely heavy drinkers (more than 60 g/day of alcohol); (12) participants who use medicines or who habitually consume health-promoting foods; (13) participants who plan to engage in activities that affect skin condition at measured sites during the study; and (14) others who have been determined as ineligible for the research participant of this study, judging from the principal investigator’s (physician’s) opinions on the findings of their background and other relevant factors. The principal investigator confirmed participants' eligibility for the trial based on background information and medical examination results before enrollment. For sample size estimation, assuming a type I error (α) of 0.05, type II error (β) of 0.8, an expected difference (δ) of 2, a standard deviation (σ) of 3, and a 1:1 group ratio, the required sample size was calculated as 72 (36 per group) using EZR (version 1.40) (Saitama Medical Center, Jichi Medical University, Saitama, Japan) [28]. Accounting for potential dropouts, the target sample size was set at 80 (40 per group). No interim analysis was planned.

Test food

For this trial, the Sph tablet and placebo tablet were produced and packaged in plain aluminum foil bags. The Sph tablet contained 23.6 mg of dairy-derived Sph (ceramide-phosphocholine) per daily intake (six tablets). The Sph tablet was combined with a Sph-rich dairy product, Milei®70HPL (Milei GmbH, Leutkirch im Allgäu, Germany), as a source of Sph. The nutrient composition per six tablets was as follows: energy, 27.5 kcal; fat, 0.4 g; protein, 1.1 g; carbohydrates, 4.9 g. In the placebo tablet, Milei®70HPL was replaced with dextrin.

Intervention

After Institutional Review Board (IRB) approval, the details of this trial were fully explained to the participants according to the informed consent form, and written informed consent was obtained from all participants. Subsequently, eligible participants were enrolled and randomly assigned to either the placebo or the Sph group. During the intervention period, the participants ingested six tablets of the test food per day by licking or chewing, and recorded their test food and medication intake, physical condition, and lifestyle habits in their diaries. Participants were instructed not to change their lifestyle or skin care habits throughout the study period.

Endpoints

The primary endpoints were skin hydration and TEWL. The secondary endpoint was the subjective perception of skin hydration.

Safety assessment

Any unfavorable and unintended signs, symptoms, or diseases observed during the intervention were defined as adverse events (AEs). AEs were evaluated by the principal investigator based on participants’ diaries, self-reports, and medical interviews. Events that could not be denied to have a causal relationship to the ingestion of the test food were designated as adverse drug reactions.

Measurement of skin barrier function and subjective skin hydration

Skin hydration levels were measured on the upper arm, forearm, lower leg, and dorsum pedis, and TEWL was measured on the forearm and dorsum pedis. All measurement points were located on the left side of the body. Measurements were taken before the intake of test foods (week 0) and at six and 12 weeks after the start of ingestion (week 6 and week 12). Participants were prohibited from taking hot spring baths for two weeks and from shaving body hair at the assessment sites for one week prior to the measurement time point. Measurements were performed under standardized room conditions (temperature: 21 ± 2℃; humidity: 50 ± 10%) at the same time of day at all time points. Participants were acclimated to these conditions for at least 20 minutes prior to measurement. Skin hydration was assessed six times at each time point using a Corneometer CM 825 device (Courage + Khazaka Electronic GmbH, Cologne, Germany), and the mean of the four middle values was used as the skin hydration level. TEWL was assessed four times at each time point using a handheld Tewameter TM300 device (Courage + Khazaka Electronic GmbH, Cologne, Germany), and the average of the measured values was used as the TEWL. The devices are validated and widely used for measuring skin barrier function and skin conditions [29,30]. Subjective skin hydration on the face was evaluated at weeks 0 and 12 using a seven-point Likert scale ranging from “very bad (-3)” to “very good (+3).”

Randomization, allocation, and blinding

The test foods were identical in appearance, smell, taste, and packaging. A third-party allocation manager confirmed their indistinguishability before allocation and at the time of code-breaking. The allocation manager generated a computer-based allocation table with a block size of four and assigned consecutive numbers to the test foods according to the table. The allocation table was kept in an opaque, tightly sealed envelope and remained sealed until code-breaking. Participants were numbered by the investigators and were assigned to the test food corresponding to their assigned number. All investigators and participants remained blinded until the code was broken. Code-breaking was carried out after the database was locked and the statistical analysis plan finalized.

Statistical analysis

Efficacy analyses were conducted using the per-protocol set (PPS). Participants who withdrew consent or had protocol violations during the intervention were excluded from the PPS analysis. Missing data were not imputed. Student's t-test was used to assess between-group differences in skin hydration levels and TEWL, and a paired t-test was used to analyze within-group changes from week 0 to each time point for each treatment. The Mann-Whitney U-test was used to evaluate between-group differences in subjective skin moisturization scores, and the Wilcoxon signed-rank test was used to analyze differences between week 0 and week 12 for each treatment. Differences in the frequency of AEs among treatments were analyzed using Fisher's exact test. Demographic characteristics were analyzed using Student's t-tests for continuous variables. Statistical analyses were performed using JMP version 17.2 (SAS Institute Inc., Cary, NC), with p-value < 0.05 considered statistically significant.

## Results

Participants

**Figure 1:**
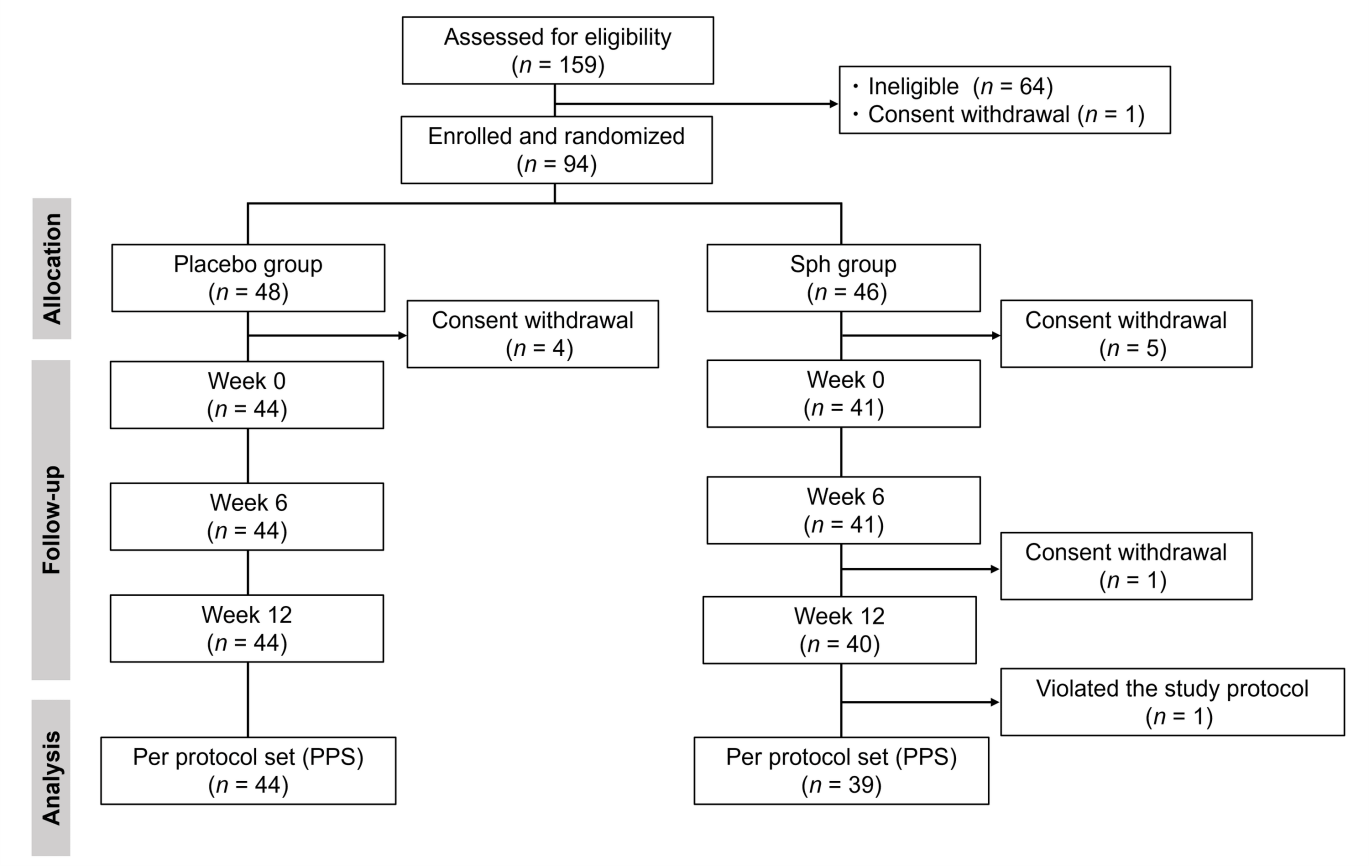
Consort flow diagram of the study Sph: sphingomyelin

Participants were recruited from October 27 to November 20, 2023. Overall, 159 participants provided written informed consent. Of these, 64 were deemed ineligible based on the inclusion and exclusion criteria, and one participant withdrew consent. Overall, 94 individuals were enrolled, and 48 and 46 of them were randomly assigned to the placebo and Sph groups, respectively. The participants started the intake of test foods, kept a diary from December 22-24, 2023, and finished on March 15-17, 2024. After the randomization, four and six participants in the placebo and Sph groups, respectively, dropped out due to consent withdrawal. Finally, 44 individuals in the placebo and 40 in the Sph groups completed this trial. Adherence was high, and the intake rate of the test foods was 99.6% in the placebo group and 100% in the Sph group. During analysis, one participant in the Sph group who violated the study protocol was excluded, resulting in 44 and 39 individuals in the placebo and Sph groups, respectively, being included in the PPS for efficacy analysis (Figure 1).

The background demographics of the two groups in the PPS were comparable in terms of age (p = 0.955), body weight (p = 0.931), height (p = 0.744), and body mass index (BMI) (p = 0.817) (Table 1). 

**Table 1:** Baseline demographics of the PPS Values are present as mean (SD) or n. p-values are results from Student’s t-test for placebo vs. Sph. p < 0.05 is considered significant. BMI: body mass index; PPS: per-protocol set; SD: standard deviation; Sph: sphingomyelin

	Placebo	Sph	p-value	
Participants, n	44	39		
Age, years (SD)	46.7 (7.4)	46.6 (7.2)	0.955	t = 0.056
Body weight, kg (SD)	53.1 (6.1)	53.2 (6.7)	0.931	t = 0.087
Height, cm (SD)	158.6 (6.2)	158.2 (5.0)	0.744	t = 0.328
BMI, kg/m^2^ (SD)	21.1 (2.4)	21.3 (2.5)	0.817	t = 0.232

Primary endpoint

**Figure 2:**
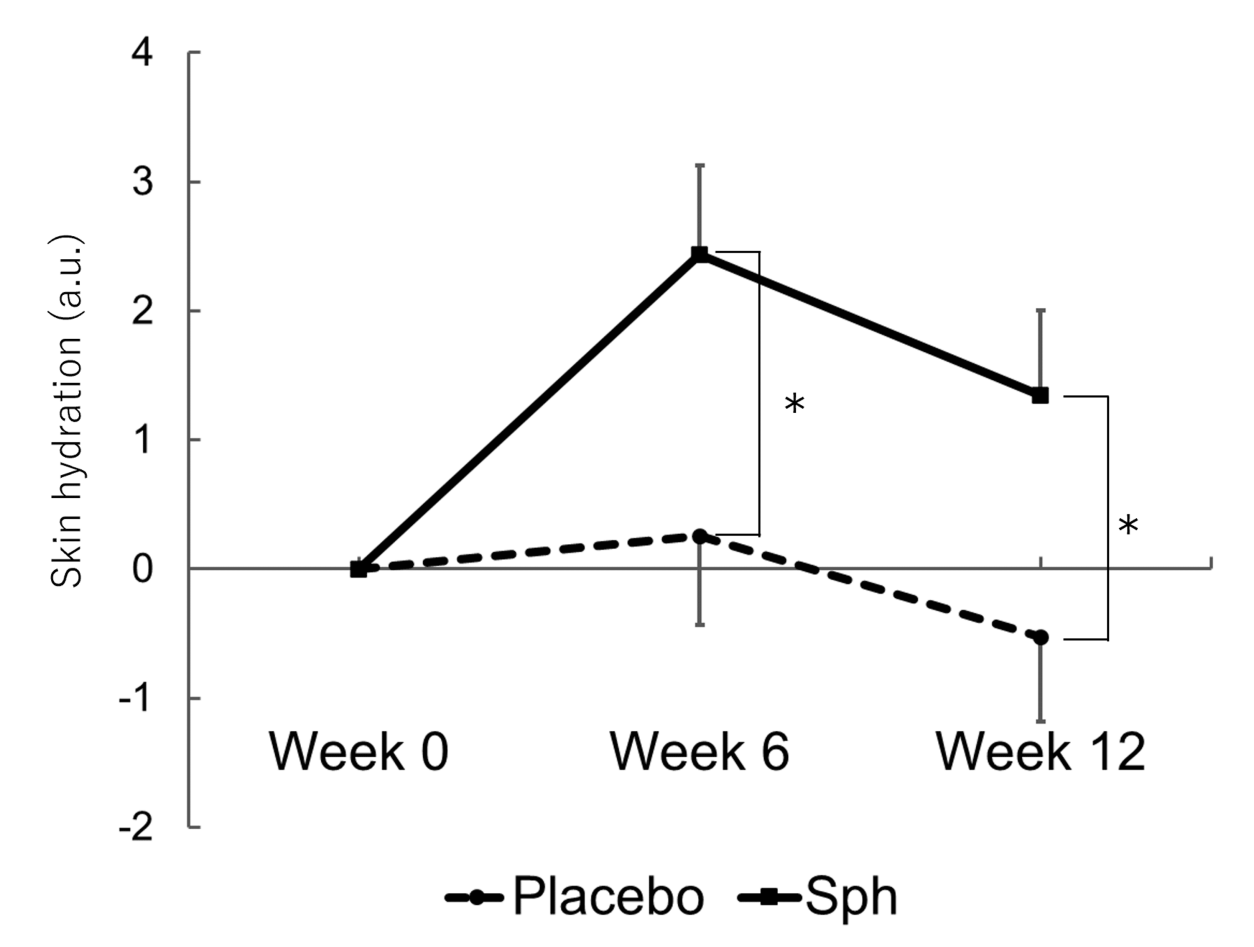
Changes in skin hydration in the upper arm The error bars represent SE (placebo n = 44 and Sph n = 39). *p < 0.05 placebo vs. Sph at each period (Student's t-test). a.u.: arbitrary unit; SE: standard error; Sph: sphingomyelin

In the placebo group, the mean increases in skin hydration levels in the upper arm from week 0 to week 6 and to week 12 were 0.3 and -0.5, respectively. In the Sph group, the mean increases from week 0 to week 6 and to week 12 were 2.4 and 1.3, respectively. The increase in skin hydration levels in the Sph group was significantly greater than in the placebo group (weeks 0-6: p = 0.017; weeks 0-12: p = 0.049) (Table 2 and Figure 2). The skin hydration levels at week 0 were comparable between the two groups at all sites. In the Sph group, skin hydration levels in the upper arm, forearm, and lower leg at week 6 were higher than those at week 0 (upper arm: p = 0.001; forearm: p = 0.041; lower leg: p = 0.006). In addition, forearm skin hydration at week 12 in the Sph group remained significantly higher than at week 0 (p = 0.047). However, in the placebo group, no changes were observed at any site at any time point compared to week 0 (Appendix A). TEWL remained unchanged from week 0 following each treatment, with no significant differences between the groups at each time point (Appendix B).

**Table 2:** Changes in skin hydration in the upper arm Values are presented as mean (SE) (a.u.). p-values are results from Student’s t-test for placebo vs. Sph at each period. p < 0.05 is considered significant. a.u.: arbitrary unit; SE: standard error; Sph: sphingomyelin

Period	Placebo (n = 44)	Sph (n = 39)	p-value	
Weeks 0-6	0.3 (0.6)	2.4 (0.7)	0.017	t = 2.446
Weeks 0-12	-0.5 (0.7)	1.3 (0.7)	0.049	t = 2.000

Secondary endpoint

Subjective perception of skin hydration significantly improved at week 12 compared to week 0 in both groups (placebo: p< 0.001; Sph: p < 0.001), but no significant differences were observed between the groups (Appendix C).

Safety assessment

AEs were evaluated in all the participants who consumed the test foods. During the intervention period, four participants in the placebo group and none in the Sph group experienced AEs; however, no case was judged to be an adverse drug reaction by the principal investigator (Table 3). Additionally, no significant differences were observed between the two groups.

**Table 3:** Adverse events during the intervention period (84 days) ^1^Represent the days from the start of ingesting test foods. ^2^A causal relationship with the intake of test foods.

Group	Age	Days^1^	Description	Relationship^2^
Placebo	50	73	Constipation	Not related
Placebo	56	73	Itchy skin	Not related
		73	Constipation	Not related
Placebo	48	13, 14, 15, 16, 17	Sore throat	Not related
		13, 14, 15, 16, 17	Chills	Not related
		13, 14, 15, 16, 17	Fatigue	Not related
		13, 14, 15, 16, 17	Headache	Not related
		13, 14, 15, 16, 17	Fever	Not related
Placebo	38	3, 28	Headache	Not related
		29, 53	Abdominal pain	Not related

## Discussion

We demonstrated that the intake of common dairy-derived Sph is safe and increases skin hydration, an indicator of skin barrier function. Overall, effects were observed throughout the body, although their appearance varied across different regions. Regarding TEWL, the measurement results did not fully correspond to the skin hydration levels.

Possible reasons why we could not detect the effects of Sph on skin hydration exclusively in the dorsum pedis may include variations in skin structure and the frequency of exposure to external stressors across measurement sites. Among the sites measured, the epidermis of the dorsum pedis was the thickest [31], and a thicker epidermis requires a longer turnover time [7]. Therefore, the intervention period in this study may have been too short to observe any effects of Sph on the dorsum pedis. Moreover, this area is often covered with socks or shoes, resulting in less exposure to external stressors, which may help maintain a healthy SC, leaving little room for improvement. A previous study investigating the effects of ceramide ingestion on skin barrier function reported that the effects tended to appear at sites exposed to the external environment [32]. Based on these considerations, the dorsum pedis - owing to its structural characteristics and sheltered condition - may not be suitable for evaluating the effects of Sph on skin condition.

The results of skin hydration levels suggested that ingestion of Sph improved skin barrier function; however, no statistically significant changes were observed in TEWL. The absence of changes in TEWL at the dorsum pedis may be attributed to the same reason why skin hydration levels at this site did not change. Regarding the forearm, although no statistically significant difference was found, TEWL at week 0 in the Sph group was lower than in the placebo group, possibly indicating limited room for improvement due to already low baseline values. Nevertheless, the decrease in TEWL from weeks 0 to 6 in the Sph group was greater than that in the placebo group. Taken together, if TEWL is measured at an appropriate site with balanced baseline values, the true effects of Sph on TEWL may become evident.

Overall, skin hydration levels increased from baseline to week 6 and then decreased from week 6 to week 12; TEWL exhibited the opposite trend. This study began in mid-December, with measurements at weeks 6 and 12 conducted in early February and mid-March, respectively. March is one of the busiest periods of the year in Japan, and the unique lifestyle habits during this time may have contributed to these data transitions.

Subjective evaluation of skin hydration did not correspond with the objective evaluation. The possibility that a placebo effect influenced the subjective assessment cannot be ruled out. In fact, participants in both groups reported a marked improvement in their perception of skin moisturization. Further efforts are needed to accurately assess this perception.

The incidence of AEs was comparable between the groups. Moreover, according to the supplier, the Sph-rich dairy product combined in the test foods has been manufactured in at least 400 tons to date as a general food ingredient. Considering these facts, the intake of dairy-derived Sph and the Sph-rich dairy product is considered safe. No AEs were observed in the Sph group. In the placebo group, four participants experienced AEs, some of which resembled infection-related symptoms. Previous clinical studies reported that ingesting MFGM-containing dairy products reduced the risk of infection [33]. As Sph is a key component of MFGM, the Sph-rich dairy product used in the test food may contain MFGM; our findings may support the previously reported roles of MFGM in biological defense.

In this study, we investigated the effects of ingesting common dairy-derived Sph on skin conditions and demonstrated that it increases skin hydration. Our findings propose a practical method for promoting skin health through the intake of common and affordable Sph-rich dairy products. In this respect, our study is novel and meaningful. 

However, this study has some limitations related to its clinical trial design. The participants were limited to healthy adults, and were homogeneous in race and gender, which may limit the generalizability of the findings. Additionally, participants were not excluded based on the type of skin care products they used, and product use during the study period was not strictly controlled, although they were instructed not to change their usual skin care habits. The differences in the products used prior to or during the study may have affected skin conditions, and it cannot be ruled out that these differences were confounding factors. Regarding outcome evaluations, the methods used in this study were limited to non-invasive measures. Histological data that could have provided insight into the mechanisms by which Sph-rich dairy improves skin hydration were not obtained. Further studies are needed to enhance internal and external validity and to clarify the underlying mechanisms.

## Conclusions

Intake of dairy-derived Sph (23.6 mg/day) increased skin hydration levels across a wide range of body sites in healthy adults. In addition, no adverse drug reactions were observed during the intervention period. Skin hydration level is an indicator of skin barrier function, which is important for maintaining skin health. Sph is a bioactive phospholipid present in milk, and some common dairy products contain high concentrations of it. Our findings provide evidence that intake of Sph-rich dairy products may represent a safe and practical method for maintaining skin health by improving barrier function.

## Appendices

Appendix A 

**Table 4:** Skin hydration levels Values are presented as mean (SE) (a.u.). ^1^Placebo vs. Sph at each time point (Student's t-test). ^2^Baseline vs. each time point following placebo treatment (paired t-test). ^3^Baseline vs. each time point following Sph treatment (paired t-test). p < 0.05 is considered significant. a.u.: arbitrary unit; SE: standard error; Sph: sphingomyelin

Site	Time point	Placebo (n = 44)	Sph (n = 39)	p-value^1^		p-value^2^		p-value^3 ^	
Upper arm	Week 0	16.9 (0.6)	15.2 (0.7)	0.060	t = 1.907	-	-	-	-
	Week 6	17.2 (0.6)	17.6 (0.7)	0.636	t = 0.476	0.662	t = 0.441	0.001	t = 3.536
	Week 12	16.4 (0.6)	16.5 (0.7)	0.903	t = 0.122	0.432	t = 0.793	0.047	t = 2.052
Forearm	Week 0	17.3 (0.7)	16.2 (0.8)	0.308	t = 1.026	-	-	-	-
	Week 6	17.4 (0.6)	18.0 (0.8)	0.561	t = 0.584	0.898	t = 0.129	0.041	t = 2.120
	Week 12	16.6 (0.7)	17.1 (0.8)	0.623	t = 0.494	0.331	t = 0.982	0.308	t = 1.032
Lower leg	Week 0	18.5 (1.2)	18.9 (1.1)	0.795	t = 0.261	-	-	-	-
	Week 6	20.0 (1.1)	21.6 (1.2)	0.323	t = 0.994	0.067	t = 1.882	0.006	t = 2.897
	Week 12	18.8 (1.0)	20.1 (1.1)	0.408	t = 0.831	0.698	t = 0.391	0.244	t = 1.183
Dorsum pedis	Week 0	20.0 (1.0)	21.1 (1.1)	0.459	t = 0.745	-	-	-	-
	Week 6	20.1 (0.8)	20.9 (1.1)	0.556	t = 0.591	0.947	t = 0.067	0.820	t = 0.229
	Week 12	20.0 (0.8)	20.0 (1.1)	0.998	t = 0.002	0.982	t = 0.023	0.163	t = 1.423

Appendix ​​​B

**Table 5:** Transepidermal water loss (TEWL) Values are presented as mean (SE) (g/h/m²). ^1^Placebo vs. Sph at each time point (Student's t-test). ^2^Baseline vs. each time point following placebo treatment (paired t-test). ^3^Baseline vs. each time point following Sph treatment (paired t-test). p < 0.05 is considered significant. SE: standard error; Sph: sphingomyelin

Site	Time point	Placebo (n = 44)	Sph (n = 39)	p-value^1^		p-value^2 ^		p-value^3 ^	
Forearm	Week 0	7.2 (0.3)	6.6 (0.2)	0.073	t = 1.817	-	-	-	-
	Week 6	6.9 (0.4)	6.1 (0.3)	0.131	t = 1.524	0.380	t = 0.887	0.131	t = 1.542
	Week 12	7.5 (0.3)	7.0 (0.4)	0.288	t = 1.070	0.435	t = 0.789	0.339	t = 0.969
Dorsum pedis	Week 0	8.0 (0.4)	7.4 (0.4)	0.307	t = 1.027	-	-	-	-
	Week 6	8.1 (0.5)	7.2 (0.4)	0.198	t = 1.298	0.733	t = 0.343	0.791	t = 0.267
	Week 12	8.6 (0.5)	8.0 (0.6)	0.419	t = 0.813	0.124	t = 1.570	0.250	t = 1.167

Appendix C

**Table 6:** Subjective perception of skin hydration Values are presented as median (IQR). ^1^Placebo vs. Sph at each time point (Mann-Whitney U-test). ^2^Baseline vs. each time point following placebo treatment (Wilcoxon signed-rank test). ^3^Baseline vs. each time point following Sph treatment (Wilcoxon signed-rank test). p < 0.05 is considered significant. IQR: interquartile range; Sph: sphingomyelin

Time point	Placebo (n = 44)	Sph (n = 39)	p-value^1^		p-value^2^		p-value^3^	
Week 0	-0.18 (0.00, 0.00)	-0.44 (-1.00, 0.00)	0.067	χ² = 3.368	-	-	-	-
Week 12	0.61 (0.00, 1.00)	0.44 (0.00, 1.00)	0.470	χ² = 0.530	<0.001	S = 358.5	<0.001	S = 294
